# Comparative Techniques of Acupuncture and Dry Needling Intersecting with Trigger Point Physiology and Diagnostics: A Cross-Discipline Narrative Review

**DOI:** 10.1089/acu.2023.0031

**Published:** 2023-10-17

**Authors:** Jordan Barber, Fabio Lodo, Andrew Nugent-Head, Xia Zeng

**Affiliations:** ^1^ AOMA Graduate School of Integrative Medicine, Austin, TX, USA.; ^2^ So Wen School of Acupuncture, Milan, Italy.; ^3^ Association for Traditional Studies, Ashville, NC, USA.

**Keywords:** acupuncture, dry needling, myofascial trigger points, trigger point physiology

## Abstract

**Background:**

Pain management is a great burden on society; therefore, cost-effective and nonaddictive treatments for pain are urgently required. Needling of painful spots has been applied in acupuncture along with dry needling <DN) to treat pain without opioids at minimal costs. However, no attempt has been made to examine DN, trigger point(TrP) physiology, and acupuncture to identify potential areas for pain-management research. This review compares the modalities of acupuncture and DN based on the current research on TrP physiology and diagnostics to advance both modalities.

**Methods:**

A narrative review of the literature on TrP physiology, its associated diagnostics, and the techniques of DN and acupuncture was performed.

**Results:**

Diagnostic imagery may benefit the study and treatment of TrPs using needling. Acupuncture and DN techniques are similar in their applications. However, the warm needling technique is established in acupuncture but not in dry needling. Additionally, translational difficulties have inhibited crossdiscipline learning.

**Conclusions:**

Historical evidence suggests a need to examine the use of heat in needling further. Additional research should be conducted on TrP categories to determine if a relationship with the needling technique can be established. Furthermore, interdisciplinary communication would benefit both modalities.

## INTRODUCTION

Pain management in the United States costs up to $635 billion^1^ annually, with 51% of adults experiencing pain at least a few days per week.^2^ Opioids are used commonly for pain management^3^; however, an average of 188 opioid overdose–related deaths were reported daily^4^ in 2020. Therefore, identifying nonaddictive and cost-effective pain treatments is critical.

Acupuncture and dry needling (DN) use filiform needles to alleviate pain.^5,6^ Myofascial trigger points (TrPs), an often overlooked cause of pain found in myofascial pain syndrome (MPS), are hyperirritable spots within the muscles that cause pain and dysfunction.^7,8^ Needling TrPs with filiform needles is a cost-efficient, opioid-free method used in acupuncture and DN to treat acute and chronic pain.

This review connects the domains of DN, TrP physiology, and acupuncture; compares the needle techniques of DN and acupuncture; identifies potential areas for pain-management research; and helps improve needling methodology by exploring the similarities and differences in needling techniques and the relevance of TrP physiology and diagnostics.

Furthermore, this literature review was conducted with the aim of influencing nonpharmacologic pain management positively by identifying research gaps in acupuncture and DN. As no such study has been conducted previously, the findings of this review will inform future research and advance knowledge in this area.

## METHODS

To identify relevant literature on TrP physiology, DN technique, and acupuncture needle technique, the databases of PubMed,^*^ Google Scholar,^†^ and Trip Database^‡^ were searched in November 2022, as they contain literature from various sources, giving a breadth of range to the search. Systematic reviews, primary research studies, literature reviews, and scholarly discussion articles were included.

### Data Collection

A Boolean search was conducted, using the following search terms: “trigger point” AND “dry needl*” OR “trigger point” AND assessment OR acupuncture AND technique OR “dry needl*” AND technique NOT case study OR case series OR case report OR 4ffect* OR efficacy OR treatment OR survey OR compar*. The key words were limited to the titles only; negative key words narrowed the results. Box 1 outlines the inclusion and exclusion criteria. The first author (J.B.) performed the search through a single-screening process using Covidence review management software.^§^

**Box 1:** Inclusion and Exclusion Criteria

Inclusion criteria	Exclusion criteria
• Written in English • Published within the last 10 years • Literature reviews • Scholarly articles • Primary research • Focuses on the musculoskeletal system • Historically or theoretically relevant • Focuses on the physiology of TrPs or the changes in physiology from intervention • Focuses on needle technique • Full article accessible • Human participants	• Comparative modalities • Results or outcome-only studies • Case studies, case reports, or case series • Focuses on the use of injectable substances • Focuses on the use of electrostimulation

TrPs, trigger points.

Publicly available books on DN, including textbooks, training manuals, or course books,^7–9^ were retrieved through Google.com, Amazon.com, and BookFinder.com using the search terms dry needling book or dry needling manual.

The *Journal of Chinese Medicine'*s (JCM's) acupuncture-technique archives were manually reviewed, using the same inclusion and exclusion criteria. Finally, the *Huang di Neijing* (
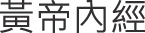
; *Yellow Emperor's Inner Classic*)—including the *Su Wen* (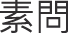; Simple Questions) and *Ling Shu* (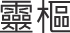; Spiritual Axis)^9^—a classical text on acupuncture needling and theory—and research articles that aided in translational accuracy were also included. The selected items discovered by backward “snowballing” references from the included items were accessed later for clarity and as additional items for statement accuracy. Items older than 10 years discovered during backward snowballing were included if they were relevant.

## Analysis

**FIG. 1.**
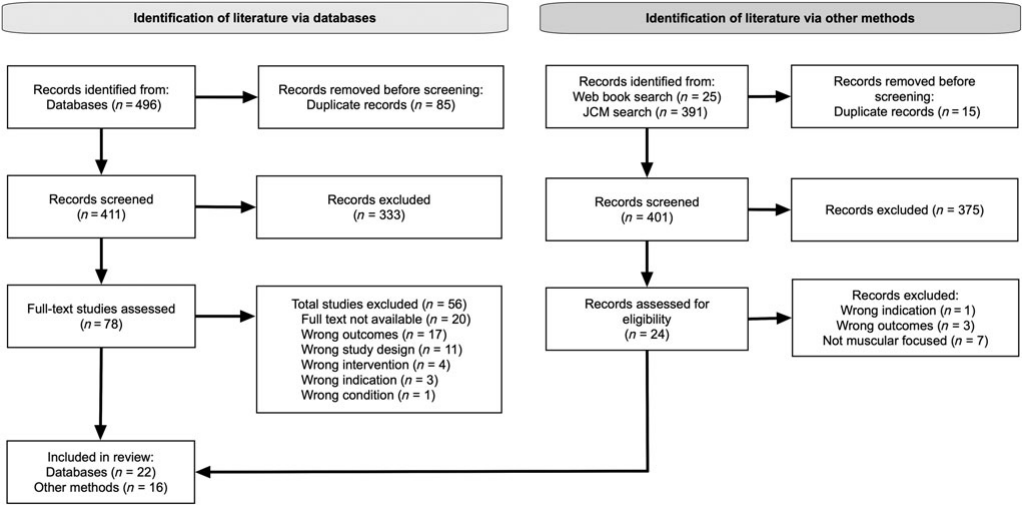
PRISMA [Preferred Reporting Items for Systematic Reviews and Meta-Analyses] flowchart. JCM, *Journal of Chinese Medicine*.

The search yielded 496 results. After removing duplicates (*n* = 85), the titles and abstracts of the remaining (*n* = 411) records were screened using the inclusion/exclusion criteria shown in Box 1 and Figure 1. Subsequently, the full text of the remaining items (n = 78) was screened, and 22 items were included after further exclusions (*n* = 56).

The book and training manual search yielded 25 results. After removing duplicates (n = 15), the titles and summaries of the remaining records (*n* = 10) were screened using the inclusion/exclusion criteria. After excluding 1 item, the remaining items (*n* = 9) were screened based on their tables of contents and a cursory review. One item was excluded subsequently; thus, 8 items were included from that search.

Reviewing the JCM technique archives yielded 391 related articles. The titles and abstracts of the results were screened, resulting in exclusions (*n* = 374). Full-text reviews were performed subsequently, resulting in additional exclusions (*n* = 9). Eight articles were included from the JCM search.

Thus, 38 items were included in this review.

## RESULTS

### DN Techniques

The 6 training manuals identified were reviewed to codify the current methods of DN techniques. The techniques were extracted (Table 1) and categorized by the needle-insertion depth (superficial or deep), and similar techniques were combined, resulting in 11 techniques categorized by primary clinical focus, using their generalized clinical application (Table 2).

**Table 1.** Dry Needle Techniques Identified

Source 1st author, yr & ref.	Technique	Clinical purpose	Description of technique
Dommerholt, 2018^8^	SDN	TrP	Insert needle superficially into TrP, do no stimulation; remove needle after 30 sec.
	DDN	TrP	Insert needle intramuscularly into TrP.
	Gunn-IMS	Neuropathic myofascial pain	DDN taut bands & tender motor points.
	*Fu* Subcutaneous	Soft-tissue injuries	Perform subcutaneous needling, stimulation, & long retention of needle (it is considered acupuncture).
	Eliciting local twitch response	TrP	Needle muscle for twitch response.
	Rotation	Fascia	Rotate the needle.
	Piston	Fascia	Lift & thrust needle.
	Percutaneous needle tenotomy	Tendinopathy	Use bevel of needle to section tendon.
	Tendon fenestration	Tendinopathy	Repeat insertions into a tendon.
	Eliciting global twitch response	Spasticity	Needle spastic muscle for whole muscle contraction.
Optimal Dry Needling Solutions, 2017^48^	SDN	Pain & fascia	*Pain:* Twirl & remove after 30–60 sec. *Fascia:* wind & retain for 15–30 min.
	DDN	TrP	Not defined.
Sharkey, 2017^32^	Eliciting local twitch response	TrP	Lift & thrust needle for twitch response.
Gyer, 2016^13^	Tendon techniques	Tendinopathy	Use multiple acupuncture techniques & multiple needles.
	Muscle technique	TrP	Use acupuncture techniques: local insertion; distal insertion; & needling of painful spots.
Haynes, 2022^49^	Piston	TrP	Piston until local twitch response cannot be elicited.
	Rotation (bidirectional, unidirectional)	TrP, fascial mobilization	Rotate to elicit local twitch response, unidirectional for mobilizing tissue.
Donnelly, 2019^7^	DDN into TrP	TrP	Insert into located TrP with or without twitch response.

yr, year; SDN, superficial dry needling; TrP, trigger point; sec, seconds; DDN, deep dry needling; Gunn-IMS, Gunn intramuscular stimulation; min, minutes.

**Table 2.** Dry-Needle Techniques Categorized

Primary category	Primary techniques	Primary focus
Superficial	*Fu* subcutaneous	Soft-tissue injury
	Superficial needling	TrP
	Multiple needles (proximal &/or distal)	Pain
Deep	Local twitch response	TrP
	Global twitch response	Spasticity
	Gunn-IMS	Neuropathic pain
	Rotating or twisting	Fascia
	Tendon-needling techniques	Tendinopathy
	Lifting & thrusting (piston)	TrP & fascia
	Deep needling	TrP
	Multiple needles (proximal &/or distal)	Pain

TrP, trigger point; Gunn-IMS, Gunn intramuscular stimulation.

### Acupuncture Techniques

The primary source of acupuncture techniques, the Spiritual Pivot,^9,10^ was reviewed, and 15 needle techniques most closely associated with work on musculoskeletal pain were extracted (Table 3). The Spiritual Pivot lists 26 acupuncture techniques divided into 3 categories in chapter 7: *Nine piercings*; *Twelve regulations*; and *Five depots*. In each category, 4/9 piercings, 8/12 regulations, and 2/5 depots were associated with direct musculoskeletal pain treatment (Table 3). The techniques related to organ pain or distal pain treatment through acupuncture-channel regulation were excluded.

**Table 3.** Acupuncture Techniques^9^

Technique	Category	Translation	Purpose	Brief summary
*jing ci* 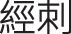	Nine piercings	Channel needling	Pain	Needling areas of hardness or pain (not specific points)
*fen ci* 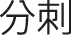	Nine piercings	Divide needling	Pain	Needling deeply between the muscle layers
*mao ci* 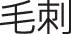	Nine piercings	Hair needling	Numbness & pain	Superficial needling between the skin & derma
*cui ci* 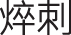	Nine piercings	Glowing needling	Pain gets worse with cold	Heated needle is inserted into the painful spot
*bao ci* 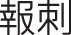	Twelve regulations	Repeated needling	Pain without a specific location	Inserting needle, retaining, then inserting another needle after eliciting pain
*hui ci* 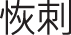	Twelve regulations	Extended needling	Tendinopathy/Sinew-channel	Needling the Sinew-channel in multiple directions to free up restrictions
*qi ci* 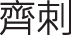	Twelve regulations	Gathered needling	Pain	Multiple needles inserted into one location
*yang ci* 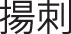	Twelve regulations	Spreading needling	Pain in a large area	One needle is inserted deeply with shallow needles surrounding it
*zhi zhen ci* 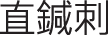	Twelve regulations	Straight needling	Pain	Parallel insertion into the superficial layers of the skin
*duan c*i 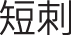	Twelve regulations	Bone needling	Deep pain	One needle is inserted deeply & then pistoned, tapping the bone
*fu ci* 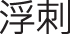	Twelve regulations	Floating needling	Muscular contraction	Oblique superficial needling
*bang ci* 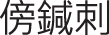	Twelve regulations	Sideways needling	Chronic pain	One needle is inserted perpendicularly; a second needle is inserted obliquely at the angle of the tip of the first needle
*guan ci* 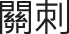	Five depots	Articulation needling	Tendinopathy/Sinew-channel	The needles are inserted perpendicular to the sides of the Sinew-channel
*he gu ci* 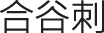	Five depots	Valley needling	Pain	Inserting, withdrawing superficially, changing the angle, & inserting deeply again; repeat in multiple directions

### TrPs and Chinese Medicine

The relationship between TrPs and acupuncture points has been explored for more than 50 years.^11^ Travell and Simon's manual on TrPs discusses the possible correlation,^12^ thereby establishing a historical relationship between TrPs and acupuncture that is often cited in reviews.^6,13–22^ Painful spots to be needled, regardless of their correlations with established acupuncture points, were called *Ashi* (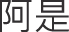) point by the physician Sun Simiao in the early sixth century in China.^23,24^

The needling of points located by tenderness on or off of an established acupuncture channel is recommended in the Spiritual Pivot,^23^ a text dated between the second and first centuries bce.^9^ *Ashi*, translated as *“ah! yes”* in Chinese Medicine (CM) lore, indicates that *“right here is the pain.”* This historically signifies the commonality of using painful spot needling, as needling *Ashi* points during the Three Kingdoms time (220–280 ce) had an ambiguous meaning—indicating use for an important minister in the imperial court as well as for the common folk. However, needling *Ashi* was used by both common folk and the imperial minister due to its popularity and simplicity, thereby alluding to the high quality of the technique.^24^ The treatment of *Ashi* points is an established technique still in practice.

A 2022 review found that “the 255 most common trigger points illustrated in the first edition of the *Trigger Point Manual* are fundamentally similar to classical acupuncture points. … ”^25^ Moreover, a 3-part study^26–28^ reported an anatomical correlation of 93.3%.

In CM, *Ashi* points are formed when the Qi and Blood cannot flow within a region owing to trauma, overuse, postural issues, climatic factors, or metabolic issues.^23^

### TrP Diagnostics

A 2021 systematic review of the assessment of myofascial TrPs via imaging concluded that a distinct change in blood flow in TrPs is observed on Doppler imaging and that active TrPs (A-TrPs) and latent TrPs (L-TrPs) are quantifiably distinct from healthy tissues.^14^ The pulsatility index and retrograde diastolic blood flow are higher, with an increased peak systolic velocity (PSV) and decreased minimum diastolic velocity (MDV) within A-TrPs than those of L-TrPs. A-TrPs create pain or dysfunction without elicitation; in contrast, L-TrPs require elicitation to create pain or dysfunction. PSV and MDV can differentiate between A-TrPs and L-TrPs.^14,29,30^

It was also revealed that diagnostic methods, including ultrasound (US), magnetic resonance imaging, and infrared thermography, may also detect TrPs; however, further extensive clinical studies are warranted to test the accuracy of these methods of diagnostic testing. Similarly, artificial intelligence modeling with surface electromyography may detect MPS.^31^ Table 4 provides an overview of the diagnostic methods reviewed.

**Table 4.** Trigger Point Diagnostics

Method	Indication
Ultrasound—grayscale (B-Mode)	TrPs are hypoechoic with heterogeneous echotexture^15^
Sonoelastography	*Doppler Vibration sonoelastography:* 100 Hz visualizes stiff tissue regions as a decrease in peak vibration & *color Doppler* shows color variance^14^
	*Shear-wave sonoelastography:* Increased shear-wave speeds & velocity in the TrP muscle tissue^15^
Magnetic resonance imaging	Focal single alterations (higher-intensity T-2 signal)^15^
Magnetic resonance elastogram	Tissue stiffness presented in 2-color–dimensional elastograms^15^
Infrared thermography	Increase in temperature at a TrP of 0.8–1.5°C^15^
Doppler ultrasound	TrPs show alterations in blood flow^15^
	*A-TrPs:* higher PIs & retrograde blood flow & 69% retrograde diastolic flow; PSV increased; MDV decreased^29^
	*L-TrPs:* 16.7% retrograde diastolic flow^29^
SEMG	Multiscale wavelet energy variation model for SEMG with sensitivity of 53.85% & specificity of 83.33% for identifying myofascial pain^22,31^

TrPs, trigger points; A-TrPs, active trigger points; PI, pulsatility index; PSV, peak systolic velocity; MDV, minimum diastolic velocity; L-TrPs, latent trigger points; SEMG, surface electromyography.

### DN's Mechanism of action

A literature review revealed that, although the mechanism of action of DN remains unclear, it is widely acknowledged that DN involves the interplay of multiple biochemicals on the sarcomeres of the muscle tissue and their impact on the neural pathways and the sodium–potassium pump, leading to alterations in muscular polarization.^7,13,22,32,33^

A neurologic component to needling has been suggested.^22^ For instance, the local twitch response is a spinal-cord reflex^22^ that can reduce the levels of neurotransmitters in the surrounding tissue, such as substance-P, calcitonin gene-regulating peptide, and inflammatory glycoproteins and cytokines when elicited.^22^

Sharkey^32^ discussed the biotensegrity of the fascial system. The role of needling in inducing mechanotransduction in the surrounding tissue, adjusting the tonality of the connective tissue, and changing its biochemical matrix, is emerging. The theory of biotensegrity states that the human body is a seamless network of interconnected tissues. Thus, the dynamic interactions between individual cells and the extracellular matrix are integral components of the overall fascia system. This approach emphasizes the interconnected nature of the entire body and recognizes the communication and balance present within this dynamic system.^34^

### Acupuncture's Mechanism of Action

The historical mechanism of action of acupuncture is based on its effect on Qi (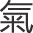). Qi, often translated as *vital energy* or *life force*, is actually theoretically complex and not easily translated. In medicine, Qi can be best described as the “metabolism” seen in the metabolic relationship and homeostasis that a unit, structure, or region of the body has with another one.^35–38^ Notably, the needling sensation of De Qi (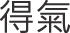; obtaining Qi) or Qi Zi (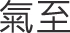; the arrival of Qi) is the principal focus of acupuncture.^9^ This is the mechanism that establishes homeostasis, which is known as making the area *zheng* (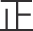; upright/correct). However, as this article focuses on the effects of needling, a detailed explanation of the acupuncture theory is beyond its scope.

The physiologic effects of acupuncture and DN are identical in musculoskeletal needling,^6,39^ despite the differences in language and the models used in acupuncture theory. The clinical mechanisms are identical, regardless of the cultural or linguistic paradigm used.

## DISCUSSION

This review compared the needle techniques utilized in acupuncture and DN and determined the similarities and differences between the two modalities. The findings indicated that there are parallels between these methods, with acupuncture involving a more-diverse approach for needling in pain management not yet explored in the domain of DN. Furthermore, the literature was reviewed to determine if any guidance was available for needling techniques based on the studies on TrPs, and parallels were identified between CM theory and categories of TrPs. This review also highlighted the need for increased communication and collaboration between the disciplines of acupuncture and DN and identified areas for future research at the intersection of acupuncture, DN, and TrP studies.

### Need for Communication

This review revealed 2 areas of miscommunication between the fields of DN and acupuncture: (1) misunderstanding of acupuncture theory and the complexity of translating it into Western science and (2) controversy regarding both DN and acupuncture as modalities.

#### Translational difficulties

The historical and cultural framework that developed CM hinders translating the complex philosophical terms often expressed in poetic observational and relational language that does not often pair with similar understandings as that of its Western anatomical counterpart.^40,41^ Attempts at introducing Western medical terms to represent CM illnesses often remove theoretical concepts used to diagnose and treat illnesses in CM and introduce foreign concepts that do not integrate within the CM framework.^42^

This review suggests that CM reasoning is analogous to script theory,^43^ making use of goal-directed knowledge to enable easy performance of tasks, which, when used within medicine in the form of illness scripts, can improve clinical reasoning by organizing medical knowledge.^44,45^

These translational issues often constitute the controversy between dry needling and acupuncture. The dismissal of the CM theory, which is foreign to Western thought, is a form of Western medical colonization and cultural imperialism that imperils the progress and health of cultural medicine and its people.^46^ Thus, a collaboration between different medical systems is required to establish common ground and advance clinical outcomes.

Additionally, since the 1900s, the term *dry needle* (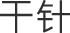: *gan zhen*) was also used for acupuncture^47^ confounding any delineation of the 2 modalities further.

#### Controversy

Several proponents of DN downplay the significance of CM theory, including acupuncture, dismissing it as superstition, unscientific, and based on mysterious energetic systems^6,48,49^; however, this outlook is predominant in the United States^17,18^ and may stem from the political and economic nature of the scope of practice and the issues related to it.^19^ Some researchers recognize the influence of acupuncture on DN but state that myofascial TrPs are not acupuncture points as they do not always correlate with known acupuncture points located on the acupuncture channels,^32^ which is opposite the historical use of nonchannel points (*Ashi* points). Yet, in contrast, TrPs have been significantly correlated in some studies,^5,25–28^ exemplifying the need for further crossdisciplininary communication.

There are discrepancies in the training and supervision requirements for DN in the United States,^50^ which may confound these issues. Furthermore, the practice of DN is becoming increasingly similar to that of acupuncture in terms of the duration of needle insertion and the number of needles required for treatment,^6^ which may result in disagreements between proponents of both modalities. The American Medical Association (AMA)

recognizes dry needling as an invasive procedure and maintains that dry needling should only be performed by practitioners with standard training and familiarity with routine use of needles in their practice, such as licensed medical physicians and licensed acupuncturists.^51^

### Further Research

Further inquiry into areas of TrP imagery and needle technique may be beneficial to enhancing clinical evidence and outcomes.

#### Diagnostic imagery

The use of diagnostic and locational imagery can improve the clinical outcomes of MPS, as the location of the TrPs is currently based on subjective palpation.^52^ US is a safe and cost-effective procedure that is being researched in injection-based therapies^52–54^ and emerging in DN to track changes^55^ in TrP with treatment. Further studies must explore the clinical outcomes of using US in locating and differentiating TrPs or *Ashi* points and confirming their resolution through DN and acupuncture.

Literature regarding differentiation of the TrP categories, active versus latent, and if this affects choice of needle technique during treatment is lacking. This differentiation is possible through diagnostic imagery and palpation, and should be studied further.

#### Needle technique

Various DN techniques are used to treat musculoskeletal pain based on the target tissue and confounding factors. Needle insertion and lifting/thrusting with localized twitch responses are used commonly (Tables 2 and 3) for TrPs and muscular pain, with some stylistic variations. However, a 2021 systematic review^15^ showed inconsistencies in technique reporting and a lack of adherence to clinical guidelines, calling for the adoption of the STandards for Reporting Interventions in Clinical Trials of Acupuncture (STRICTA)^56^ for further investigation.

Acupuncture techniques for musculoskeletal pain are differentiated based on the location, depth, extent, chronicity, and temperature influence, which are absent in DN.

In contrast, DN often seeks a localized twitch response via pistoning needling; however, acupuncture with oblique insertion without manipulation, retention, and subsequent withdrawal when the needling sensation dissipates is clinically effective,^39^ and mirrors many techniques from the Spiritual Pivot.

The extending technique, a classical technique in acupuncture, can be associated with a twitch response, which may indicate a muscular contraction.^#^

It is imperative to discuss De Qi more as it is the principal component of efficacy in acupuncture. According to Kong et al.,^57^ Chapters 1, 3, and 9 of the Spiritual Pivot emphasize the importance of De Qi within acupuncture.

The definition of De Qi is debated.^57–59^ De Qi has been described as sensations of soreness, numbness, fullness or distension, and heaviness^59–61^ with feelings of temperature changes, pain, itching, muscular twitching^59^ and changes in facial or body expression. The academic debate centers on whether De Qi is any sensation a needle elicits or includes sensations the acupuncturist experiences as the needle engages with the tissue.

Acupuncture emphasizes sensation, unlike DN, and the correlation between eliciting sensation and the effectiveness of acupuncture has been shown.^61^

Further dialogue and research should explore the topic of needle sensation within acupuncture and DN to establish effective clinical guidelines.

Table 5 presents the parallels observed between the established DN and classical acupuncture techniques.

**Table 5.** Parallels Seen in Acupuncture and Dry-Needle Techniques

Primary category	Techniques	Correlations
Superficial	Hair needling	SDN, *Fu*
	Straight needling	SDN, *Fu*, Pist
	Floating needling	SDN, Pist
Deep	Channel needling	SDN, DDN, Rot, LTR, Gunn-IMS, Pist
	Divide needling	DDN, Pist
	Glowing needling	No correlation
	Extended needling	LTR, Ten
	Gathered needling	MN
	Bone needling	DDN, Pist
	Articulation needling	Ten
	Valley needling	SDN, DDN, Rot, LTR, Pist
	Sideways needling	MN
Mixed	Spreading needling	MN
	Repeated needling	MN

SDN, superficial dry needling; *Fu*, *Fu* (subcutaneous); Pist, piston (lifting and thrusting); DDN, deep dry needling; Rot, rotating or twisting; LTR, local twitch response; Gunn-IMS, Gunn-intramuscular stimulation; Ten, tendon needling; MN, multiple needles.

##### Target differentiation

The differences between the biochemicals^7,13,22,32^ and blood flow^14,29,30^ in A-TrPs and those in L-TrPs and in acute and chronic injuries provide a basis for differentiating needling approaches, potentially reducing pain and postneedling soreness often cited^6,16,20,22^ as a side-effect of DN but minimally in acupuncture. Acupuncture parallels this differentiation based on the theory of Qi and Blood; however, this is not observed in DN, in which TrP type is not a factor. Future research should explore needle techniques, TrP categories, and the correlation between the CM theory and the nature of TrPs.

##### Temperature techniques

According to CM, weather and temperature, especially Cold, can create or worsen pain. The mechanism of action may contrast with modern Western scientific views; however, Cold^62^ and Heat^63^ have clinically relevant effects on tissue microcirculation. Acupuncture treats Cold-induced pain by heating and inserting a hot needle (glowing needle technique). Although not researched in the West, heating a needle for musculoskeletal pain may induce unique biochemical changes with clinical outcomes and be a primitive form of percutaneous thermablation, which destroys cancer tissue with high temperatures but differs in targets, applications, and sustained high temperatures.

### Limitations

The analytical generalizations of this review may be limited by the comparison of qualitative data and potential errors of scrutiny or overgeneralizations during inductive and conformity reasoning.^64^

This narrative review, as a form of secondary research evidence, was conducted to provide interpretation and critique and is not suitable for direct clinical changes. This review focuses on clinical modalities and is not exhaustive historically.

Due to the lack of global standardization in DN training, the reviewed material includes varied sources, including those from physical therapists and MDs, both domestically and internationally. The foundational knowledge base of these sources should be considered in assessing its validity. 

## CONCLUSIONS

This review identified areas for further exploration in nonpharmacologic pain management, encompassing myofascial pain, acupuncture, and DN. Future studies should investigate the correlation between TrP categories and CM theory to improve needling techniques. Crossdisciplinary discussions between acupuncture and Western medicine could help develop a more-comprehensive pain management model. Innovative techniques, such as diagnostic US, could improve outcomes and increase objectivity. Thus, further well-designed randomized clinical trials are required in this area.

## Notes

^*^
Visit: https://pubmed.ncbi.nlm.nih.gov for more information.

^†^
Visit: https://scholar.google.com for more information.

^‡^
Visit: https://www.tripdatabase.com for more information.

^§^
Visit: https://www.covidence.org for more information.

^#^
“, , , , , ” can be translated as “The third is called extended needling. Extended needling, needle close to the affected area, lifting and thrusting (pistoning) in front and behind/around the affected area, restoring sinew channel contraction/quickly, this is how to treat obstruction in the sinew channels.” Although  is often not translated in English translations as a twitch response, it is believed that this may mean the area that is stimulated contracts.
